# Re-evaluation of lymph node dissection and surgical strategy for locally advanced gastric cancer in the era of neoadjuvant immunotherapy: a narrative review

**DOI:** 10.3389/fonc.2026.1924559

**Published:** 2026-09-23

**Authors:** Yuzhou Mei, Yipeng Cui, Huiming Wu, Songhao Liu, Liang Xue, Huiyu Li

**Affiliations:** Department of General Surgery, Third Hospital of Shanxi Medical University, Shanxi Bethune Hospital, Shanxi Academy of Medical Sciences, Tongji Shanxi Hospital, Taiyuan, Shanxi, China

**Keywords:** gastric cancer, immunotherapy, locally advanced gastric cancer, lymph node dissection, surgery

## Abstract

Gastric cancer is a highly prevalent malignancy with high mortality worldwide. Radical surgical resection remains the cornerstone for achieving long-term survival in patients with locally advanced gastric cancer. In recent years, immune checkpoint inhibitors have been increasingly introduced into the perioperative setting. In locally advanced gastric cancer, immunotherapy shows potential to improve pathological complete response rates and R0 resection rates. However, its overall survival benefit still requires confirmation from high-quality studies. Meanwhile, immune-related inflammatory responses in tumors and lymph nodes induced by immunotherapy may increase uncertainty in imaging evaluation. This complicates preoperative staging and intraoperative assessment. Against this background, whether the extent of D2 lymph node dissection and surgical strategy should be individualized based on response to neoadjuvant immunotherapy remains controversial. This review summarizes recent advances and ongoing controversies regarding surgical strategies and the extent of lymph node dissection in locally advanced gastric cancer in the era of neoadjuvant immunotherapy, aiming to provide references for clinical decision-making.

## 1 Methodology

This narrative review was conducted to summarize current evidence regarding neoadjuvant immunotherapy, lymph node dissection, and surgical strategies for locally advanced gastric cancer. A literature search was performed in PubMed, Web of Science, Embase, and the Cochrane Library for studies published up to June 2026. The search terms included combinations of “gastric cancer,” “gastroesophageal junction cancer,” “locally advanced gastric cancer,” “neoadjuvant immunotherapy,” “perioperative immunotherapy,” “immune checkpoint inhibitor,” “PD-1,” “PD-L1,” “lymph node dissection,” “lymphadenectomy,” “D2 gastrectomy,” “surgical strategy,” “pathological complete response,” and “tumor downstaging.” Original clinical studies, randomized controlled trials, prospective and retrospective cohort studies, systematic reviews, meta-analyses, clinical guidelines, and relevant translational studies were considered. Priority was given to recent high-quality clinical trials and studies directly addressing pathological response, R0 resection, lymph node regression, surgical safety, survival outcomes, and the potential immunological role of regional lymph nodes. Studies focusing exclusively on unresectable or metastatic disease without relevance to perioperative or surgical management were generally excluded. Additional relevant publications were identified by manually screening the reference lists of included articles. The retrieved evidence was narratively synthesized according to three main themes: advances and controversies in neoadjuvant immunotherapy, the extent and biological implications of lymph node dissection, and response-guided surgical decision-making after neoadjuvant treatment. Owing to heterogeneity in study design, treatment regimens, patient populations, pathological response definitions, and follow-up duration, no formal meta-analysis was performed.

## 2 Background

Gastric cancer is one of the most common malignant tumors of the digestive system worldwide. It ranks fifth in incidence and fourth in mortality, with a particularly high disease burden in East Asia (1–3). Most patients are diagnosed at the stage of locally advanced gastric cancer (LAGC) (2). According to the 8th edition of the American Joint Committee on Cancer/Union for International Cancer Control (AJCC/UICC) TNM staging system, LAGC is generally defined as clinical stage cT2–4aN0–3M0 (4). In recent years, immune checkpoint inhibitors have achieved major breakthroughs in first-line treatment of advanced gastric cancer. Studies such as CheckMate 649 have provided high-level evidence and have promoted the extension of immunotherapy to the perioperative and stage IV conversion settings (5). At present, the combination of immunotherapy and chemotherapy in LAGC is being actively explored. Although high-quality long-term survival data for perioperative immunotherapy are still lacking, early evidence suggests that neoadjuvant immunotherapy combined with chemotherapy may significantly increase the pathological complete response (pCR) rate and improve tumor downstaging and R0 resection rates compared with conventional neoadjuvant chemotherapy (6). Several pivotal clinical trials have further established the value of neoadjuvant therapy in gastric cancer. For example, the FLOT4 and RESOLVE studies demonstrated that neoadjuvant chemotherapy (NAC) can significantly improve R0 resection rates and survival outcomes, providing a strong evidence base for the application of neoadjuvant therapy (NAT) in advanced gastric cancer (7, 8). More recently, phase III perioperative immunotherapy trials, such as MATTERHORN and KEYNOTE-585, have shown encouraging pathological response trends, further supporting the role of immunotherapy in the perioperative treatment paradigm (9, 10). With the continuous evolution of treatment strategies, the application of neoadjuvant therapy in LAGC has become increasingly standardized. However, several key issues remain unresolved, including treatment cycle optimization, timing of surgery, and the management of immunotherapy-related atypical responses such as pseudoprogression and hyperprogression. Current studies suggest that surgery is generally recommended 4–6 weeks after completion of neoadjuvant immunotherapy to reduce treatment-related adverse effects and optimize surgical safety, while also allowing an appropriate window for response assessment (11). Nevertheless, no consensus has been reached on balancing maximal therapeutic efficacy with optimal surgical timing. At the same time, surgical strategies for LAGC are being re-evaluated in the context of advances in perioperative therapy. D2 lymphadenectomy has become an international standard and is recommended in guidelines according to different types of gastrectomy. However, with the increasing use of neoadjuvant immunotherapy, tumor immune microenvironment and regional lymph node immune function may be reshaped. This raises concerns that lymph node dissection standards established in the chemotherapy era may require re-evaluation. At present, whether the extent of D2 dissection should be modified in the immunotherapy era remains controversial (12). From an oncological surgical perspective, standardized and adequate lymph node dissection remains essential for achieving curative resection, improving staging accuracy, and reducing recurrence risk. However, evidence on altered lymph node response patterns after neoadjuvant immunotherapy and their impact on surgical extent remains limited. High-quality prospective data supporting systematic modification of dissection extent are still lacking. Therefore, it is necessary to systematically re-evaluate perioperative surgical strategies for LAGC in the era of immunotherapy, particularly the appropriate extent of lymph node dissection and potential optimization strategies, to achieve a balance between radical resection and individualized treatment.

## 3 LAGC and neoadjuvant immunotherapy

### 3.1 Current status and advances

Immune checkpoint inhibitors (ICIs) are currently classified into three main categories according to their targets: anti-programmed cell death-1 (PD-1) antibodies (e.g., nivolumab), anti-programmed cell death ligand-1 (PD-L1) antibodies (e.g., durvalumab), and anti-cytotoxic T-lymphocyte-associated protein 4 (CTLA-4) antibodies (e.g., ipilimumab) (13). Given the marked molecular and immunological heterogeneity of gastric cancer, single-agent immunotherapy is insufficient to benefit all patients. Therefore, combination strategies involving ICIs have become a major focus of current and future research. The main approaches include immunotherapy combined with chemotherapy, targeted therapy, radiotherapy, or dual immunotherapy regimens (14). Among these, neoadjuvant immunotherapy-based combinations have become an important component of multidisciplinary treatment for locally advanced gastric cancer (LAGC).

#### 3.1.1 Neoadjuvant immunotherapy combined with chemotherapy

Neoadjuvant immunotherapy combined with chemotherapy is currently the most widely used and best supported strategy (15). Chemotherapy can enhance immunotherapy efficacy through multiple mechanisms, including induction of immunogenic cell death, release of tumor-associated antigens, depletion of immunosuppressive cell populations, promotion of effector T-cell infiltration, and upregulation of major histocompatibility complex (MHC) expression, thereby reshaping the tumor immune microenvironment (16). Multiple clinical studies have demonstrated the advantages of this combination in pathological response and tumor downstaging. The NEOSUMMIT-01 study showed that the pathological complete response (pCR) rate was significantly higher in the combination group compared with chemotherapy alone (22.2% vs 7.4%, P = 0.03). The major pathological response (MPR) rate was also significantly improved (44.4% vs 20.4%, P = 0.009), along with improved tumor downstaging (17). The DANTE study further confirmed that immunotherapy plus chemotherapy increased the pCR rate compared with chemotherapy alone (24% vs 15%, P = 0.032), with greater benefit observed in patients with high PD-L1 expression and MSI-H status (18). The KEYNOTE-585 trial demonstrated improved pCR rates with the addition of immunotherapy in both the overall population and the FLOT subgroup (12.9% vs 2.0%; 13.0% vs 2.4%) (11). Similarly, the MATTERHORN trial reported a significantly higher pCR rate with combination therapy (19.2% vs 7.2%, P < 0.00001), as well as improvements in event-free survival (EFS) and overall survival (OS), with an OS hazard ratio of 0.78 (95% CI 0.63–0.96, P = 0.021) (9).

#### 3.1.2 Neoadjuvant immunotherapy combined with targeted and anti-angiogenic therapy

Approximately 10%–12% of gastric cancers overexpress human epidermal growth factor receptor 2 (HER2). HER2-targeted therapy has been established in advanced gastric cancer and is now being moved into the perioperative setting (19). Several studies have shown that immunotherapy combined with HER2-targeted therapy can significantly improve pathological response rates. For example, neoadjuvant camrelizumab combined with trastuzumab and CAPOX achieved a pCR rate of 21.7%–31.3%, while tislelizumab combined with trastuzumab and DOS further increased the pCR rate to 42.9%–58.3% (20). In addition, antibody–drug conjugates (ADCs) combined with immunotherapy have also demonstrated promising efficacy. In HER2-positive patients, the pCR rate was approximately 33%, with an R0 resection rate of up to 100% (21). Anti-angiogenic therapy can normalize tumor vasculature and remodel the immune microenvironment, thereby providing synergistic effects with immunotherapy. The DRAGON study showed that camrelizumab combined with apatinib and SOX significantly improved the pCR rate (18.3% vs 5.0%, P < 0.0001) (22). The NO LIMIT and Arise-F-G005 studies further indicated improvements in objective response rate and MPR, as well as survival outcomes. Moreover, multi-agent combination regimens have shown stronger antitumor activity. For example, dual immunotherapy combined with anti-angiogenic therapy and chemotherapy achieved a pCR rate of 61.9% and an MPR rate of 78.6%, along with high lymph node negativity and downstaging rates (23). However, most current evidence is derived from early-phase studies, and further high-level validation is required.

#### 3.1.3 Neoadjuvant immunotherapy combined with radiotherapy

Chemoradiotherapy combined with immunotherapy has shown strong potential for local tumor control in LAGC. Early clinical studies reported that sintilimab combined with concurrent chemoradiotherapy achieved a pCR rate of 38.2% and an MPR rate of 79.4%, with a median event-free survival of 21.1 months (24). The Neo-PLANET study showed that camrelizumab combined with chemoradiotherapy achieved a pCR rate of 33.3% and an R0 resection rate of 91.7% (25). The GAS-Jiangsu 01 study further reported pCR rates exceeding 40%, MPR rates of 81%, and an R0 resection rate of up to 100% in selected populations (26). Although this approach demonstrates high short-term efficacy, high-quality randomized controlled trials are still lacking to confirm long-term survival benefits and standardize its clinical application.

#### 3.1.4 Neoadjuvant dual immunotherapy

In the mismatch repair-deficient (dMMR) or microsatellite instability-high (MSI-H) subgroup of gastric cancer, dual immunotherapy has shown remarkable efficacy. The NEONIPIGA study reported a pCR rate of 58.6% and an R0 resection rate of 91% with nivolumab plus ipilimumab (27).

The INFINITY study further demonstrated that tremelimumab combined with durvalumab achieved a pCR rate of 60%, with 2-year progression-free survival and overall survival rates of 84.1% and 91.7%, respectively (28).

These findings suggest that this molecular subtype represents a highly immunotherapy-sensitive population and provides an opportunity for investigating response-adapted treatment strategies. However, non-operative management remains investigational and requires validation within carefully designed prospective clinical trials.

Representative clinical studies evaluating neoadjuvant or perioperative immunotherapy-based strategies for locally advanced gastric cancer are summarized in **Table 1**.

**Table 1:** Representative clinical studies of neoadjuvant or perioperative immunotherapy for locally advanced gastric cancer.

Study	Design and population	Treatment	Comparator	Key outcomes
NEOSUMMIT-01	Randomized phase II trial; 108 patients with resectable locally advanced gastric or GEJ adenocarcinoma	Toripalimab plus SOX or XELOX	SOX or XELOX	pCR: 22.2% vs 7.4%; 3-year EFS: 74.7% vs 56.2%
DANTE/IKF-s633	Randomized phase II/III trial; 295 patients included in the phase II analysis	Atezolizumab plus perioperative FLOT	FLOT	Complete pathological regression: 24% vs 15%; greater benefit in PD-L1 CPS ≥10 and MSI-H tumors
KEYNOTE-585	Randomized, double-blind phase III trial; 1,007 patients with resectable gastric or GEJ adenocarcinoma	Pembrolizumab plus perioperative chemotherapy	Placebo plus chemotherapy	Improved pCR and pathological downstaging; no statistically significant superiority for EFS or OS
MATTERHORN	Randomized, double-blind phase III trial; 948 patients with resectable gastric or GEJ adenocarcinoma	Durvalumab plus perioperative FLOT	Placebo plus FLOT	pCR: 19.2% vs 7.2%; significantly improved EFS
DRAGON IV/CAP 05	Multicenter randomized phase III trial in locally advanced resectable gastric or GEJ adenocarcinoma	Camrelizumab plus low-dose rivoceranib and SOX	SOX	pCR: 18.3% vs 5.0%; improved major pathological response and downstaging
Neo-PLANET	Single-arm phase II trial; 36 patients with cT3–4N+M0 gastric or GEJ adenocarcinoma	Camrelizumab plus concurrent chemoradiotherapy	None	pCR: 33.3%; MPR: 44.4%; R0 resection: 91.7%
NEONIPIGA	Multicenter single-arm phase II trial; 32 patients with resectable dMMR/MSI-H gastric or GEJ adenocarcinoma	Nivolumab plus ipilimumab	None	Among 29 operated patients, pCR: 58.6%; R0 resection: 100%
INFINITY	Multicenter single-arm phase II trial; 18 patients with resectable dMMR/MSI-H gastric or GEJ adenocarcinoma	Tremelimumab plus durvalumab	None	Among 15 evaluable patients, pCR: 60%; MPR: 80%

### 3.2 Controversies and challenges

However, neoadjuvant immunotherapy for gastric cancer still faces key challenges, including limited translation of efficacy into survival benefit and difficulties in patient selection. The KEYNOTE-585 trial evaluated perioperative treatment with chemotherapy plus anti-PD-1 therapy. Although neoadjuvant treatment significantly improved tumor downstaging and enabled some patients to achieve clinical complete response (cCR) or pathological complete response (pCR), postoperative adjuvant immunotherapy did not improve overall survival (OS) (11). Similarly, the ATTRACTION-05 trial also failed to demonstrate a survival benefit with postoperative adjuvant immunotherapy. These two negative perioperative trials contrast sharply with results in unresectable advanced gastric cancer (29). In advanced disease, CheckMate 649, ORIENT-16, and RATIONALE-305 all showed that immunotherapy-based combination regimens significantly improved OS (5, 30, 31). By contrast, perioperative trials that included patients undergoing curative resection (such as KEYNOTE-585 and ATTRACTION-05) did not reproduce these survival benefits (11, 29). The discrepancy between improved pathological response and limited survival benefit observed in perioperative immunotherapy trials highlights the complexity of translating immune activation into durable clinical outcomes. Several potential explanations should be considered. First, the lack of biomarker-based patient selection may have diluted treatment effects, as current biomarkers such as PD-L1 expression and MSI status do not fully capture immunotherapy sensitivity in the perioperative setting. Second, differences in treatment sequencing, timing, and duration of postoperative immunotherapy may influence therapeutic efficacy. In addition, surgical stress and postoperative immune alterations may affect immune recovery and treatment responsiveness. Variability in chemotherapy backbones, adjuvant treatment compliance, and patient populations may further contribute to heterogeneous outcomes among trials. The potential impact of lymph node-associated immune function represents an intriguing biological hypothesis; however, it remains one of several possible mechanisms and currently lacks direct clinical validation. In addition, both real-world evidence and clinical practice indicate that a subset of patients show limited response to immunotherapy, and some may even experience no benefit or hyperprogression. Immune-related adverse events (irAEs) further increase treatment complexity. Therefore, precise identification of patients most likely to benefit is a critical prerequisite for successful perioperative immunotherapy. Currently, commonly used predictive biomarkers include PD-L1 combined positive score (CPS)/tumor proportion score (TPS), microsatellite instability (MSI) status, and tumor mutational burden (TMB). However, their overall predictive performance remains limited, with insufficient sensitivity and poor consistency, which restricts their utility for perioperative decision-making. To address this issue, the development of novel molecular biomarkers has become a research priority. Previous studies have suggested that methylation at the PD-L1 K162 site is associated with sensitivity to anti-PD-1/PD-L1 therapy. The proposed mechanism is that methylation at this site may alter PD-1/PD-L1 binding affinity, thereby affecting immune escape pathways. Based on this, we propose the methylation PD-L1 ratio (MPR) as a potential predictive biomarker to further optimize patient selection for immunotherapy benefit (32).

## 4 Neoadjuvant immunotherapy and lymph node dissection

### 4.1 Current status and controversies in LAGC lymph node dissection

The 6th edition of the 2021 Japanese *Gastric Cancer Treatment Guidelines* is one of the most influential references for gastric cancer management worldwide. It provides a systematic definition of the standard extent of lymph node dissection for locally advanced gastric cancer (LAGC). (1) For distal gastrectomy, D2 lymphadenectomy includes stations No.1, No.3, No.4sb, No.4d, No.5, No.6, No.7, No.8a, No.9, No.11p, and No.12a. (2) For proximal gastrectomy, D2 dissection includes No.1, No.2, No.3a, No.4sa, No.4sb, No.7, No.8a, No.9, No.11p, and No.11d. (3) For total gastrectomy, D2 dissection includes No.1–No.7, No.8a, No.9, No.11p, No.11d, and No.12a. (4) When the tumor invades the esophagus, additional dissection of mediastinal lymph node stations No.19, No.20, and No.110 is recommended (33).

Beyond the standard D2 framework, selective extended lymph node dissection is recommended for certain high-risk stations or specific anatomical regions, including No.10, No.13, No.14v, No.16, and mediastinal nodes. The extent of dissection should be determined based on tumor location, depth of invasion, and risk of metastasis. Specifically, splenic hilum dissection (No.10) may be considered in upper gastric cancers involving the greater curvature, with splenectomy if necessary. Dissection of No.14v may be considered in distal gastric cancer with No.6 involvement. No.13 dissection is recommended when the tumor invades the duodenum. No.16 dissection may be selectively performed in patients with extensive nodal metastasis or residual risk after neoadjuvant therapy. For esophageal invasion of 2–4 cm, lower mediastinal lymph node dissection is recommended, whereas invasion ≥4 cm requires transthoracic approach with systematic dissection of upper, middle, and lower mediastinal nodes (33).

Currently, D2 lymphadenectomy remains the international standard for radical surgery in LAGC. Technically, lymph node dissection follows the principle of en-bloc resection, aiming for complete removal of the gastric mesentery, vascular sheaths, and surrounding lymphatic tissue along embryological fascial planes (12). However, the precise anatomical boundaries of en-bloc resection may vary among surgeons, which has contributed to the development and refinement of the mesenteric anatomy-based surgical concept.

Based on this concept, D2 plus complete mesogastric excision (D2 + CME) has been widely investigated in recent years. Studies suggest that this approach may enable more standardized mesenteric en-bloc resection, reduce intraoperative bleeding and tissue injury, and potentially improve long-term oncological outcomes by decreasing intraoperative tumor cell dissemination (34, 35). In addition, from a surgical quality control perspective, complete mesogastric excision may increase the number of harvested lymph nodes and improve staging accuracy, thereby facilitating more precise postoperative risk stratification.

Despite these advances, the extent of lymph node dissection from D1 to D2 or even D2+ remains controversial in LAGC (33). Three landmark randomized controlled trials conducted in Europe, including the Dutch trial (36), the MRC trial from the United Kingdom (37), and the Italian cooperative trial (38), failed to demonstrate a significant overall survival (OS) benefit of D2 over D1 dissection. However, all consistently showed higher postoperative morbidity and perioperative mortality associated with D2 dissection.

In long-term follow-up, the Dutch trial reported no significant difference in OS between the D1 and D2 groups (22% vs 28%, P = 0.34), but a significantly lower gastric cancer-related mortality in the D2 group (37% vs 48%, P = 0.01), suggesting a potential tumor-specific survival benefit of more extensive lymphadenectomy (36). The Italian study further showed no significant difference in 15-year OS, but in the subgroup with pT > 1N+ disease, D2 dissection significantly improved disease-specific survival (DSS) (51.4% vs 29.4%, P = 0.035). In contrast, in elderly patients (>70 years) and those with early gastric cancer, D1 dissection showed better DSS (39). These findings indicate that “more extensive” lymph node dissection is not always superior and should be tailored according to tumor stage and host factors.

In Asian studies, the JCOG 9501 trial demonstrated that para-aortic lymph node dissection (PAND) added to standard D2 surgery did not improve survival (40), thereby rejecting routine super-extended lymphadenectomy. However, in the JCOG 0405 study, patients with a risk of No.16 metastasis who received preoperative cisplatin plus S-1 chemotherapy followed by D2 + PAND achieved a 65% objective response rate and an 82% resection rate, with a 5-year OS of 53%, suggesting that extended dissection may still have therapeutic value in strictly selected patients (41).

The controversy surrounding extended lymph node dissection fundamentally reflects the balance between oncological benefit and surgical risk. On one hand, extended dissection increases lymph node yield, reduces stage migration (the Will Rogers phenomenon), improves staging accuracy, and may optimize postoperative treatment decisions. On the other hand, it increases perioperative morbidity and mortality, particularly in low-volume centers or among less experienced surgeons.

Notably, evidence suggests that in high-volume centers with well-trained surgical teams, perioperative mortality after D2 dissection can be consistently maintained below 2% (40–43), indicating that this procedure is safe under standardized conditions. Therefore, prognosis after gastric cancer surgery depends not only on the extent of lymph node dissection but also on surgeon experience, institutional volume, and perioperative management systems.

From a broader perspective, lymph node dissection strategy represents the integration of anatomical clearance and tumor biological behavior. The traditional D2 framework was developed in the surgery-dominant era and is primarily based on anatomical lymphatic drainage patterns and pathological experience. However, in the current era of neoadjuvant therapy, particularly immune checkpoint inhibitors, tumor and nodal biology have changed significantly. These changes include heterogeneous tumor regression patterns, immune remodeling of lymph nodes, and discordance between radiological and pathological residual disease.

Therefore, in the era of neoadjuvant therapy, the strategy for lymph node dissection in LAGC requires re-evaluation. It is necessary to maintain the oncological adequacy of D2 dissection while exploring whether a biologically guided “functional optimization” of lymphadenectomy is feasible, based on tumor response, immune status, and microenvironmental changes. Future studies should integrate multi-omics biomarkers, dynamic imaging assessment, and prospective surgical trials to redefine the optimal extent of lymph node dissection across different treatment contexts, thereby achieving a precise balance between radicality and individualized therapy. The indications, supporting evidence, potential benefits, and limitations of different lymphadenectomy strategies are summarized in **Table 2**.

**Table 2:** Evidence and current considerations regarding lymph node dissection strategies for locally advanced gastric cancer.

Strategy	Main extent	Potential Indications	Supporting evidence	Potential benefits
D1 lymphadenectomy	Early gastric cancer or selected frail patients	European trials showed lower morbidity but less extensive nodal clearance	Reduced operative risk	Generally inadequate for fit patients with LAGC
Standard D2 lymphadenectomy	Resectable LAGC	Supported by guidelines and long-term Dutch and Italian trial data	Accurate staging and regional disease control	Standard surgical approach
D2 plus complete mesogastric excision	Selected LAGC in experienced centers	Prospective and propensity-matched studies	More standardized en bloc resection and higher nodal yield	Promising, but long-term survival benefit remains uncertain
D2 plus No.10 dissection	Upper gastric cancer involving the greater curvature	Guideline-based selective recommendation	Improved clearance of splenic hilar nodes	Not recommended routinely
D2 plus No.13 dissection	Distal gastric cancer with duodenal invasion	Limited retrospective and guideline-based evidence	Additional clearance of posterior pancreatic head nodes	Reserved for selected patients
D2 plus No.14v dissection	Distal gastric cancer with suspected No.6 nodal involvement	Retrospective evidence	Possible improvement in regional control	Routine use is not recommended
D2 plus No.16 dissection	Selected patients with extensive nodal disease after neoadjuvant therapy	JCOG9501 showed no benefit for routine use; JCOG0405 suggested possible benefit in selected patients	Potential disease control in highly selected cases	Not recommended routinely
Mediastinal lymph node dissection	Gastric or GEJ tumors with esophageal invasion	Guideline-based recommendations	Improved clearance of mediastinal nodal disease	Extent depends on tumor location and invasion length
Response-guided lymphadenectomy	Highly selected responders after neoadjuvant immunotherapy	No prospective evidence	Possible reduction of unnecessary dissection	Investigational; standard D2 should not be reduced routinely

### 4.2 LAGC lymph node dissection in the era of immunotherapy

With immune checkpoint inhibitors demonstrating high-level evidence in first-line treatment of multiple advanced solid tumors and gradually becoming a standard therapeutic option, their mechanisms of action have been increasingly elucidated. Current consensus suggests that one of the core functions of immunotherapy is to activate and remodel host anti-tumor immune responses, in which regional lymph nodes play an irreplaceable hub role (44). Immune checkpoint inhibitors (ICIs) restore anti-tumor activity by blocking key immunosuppressive pathways, including programmed cell death protein 1 (PD-1) and its ligand PD-L1, thereby reactivating exhausted T cells.

From an immunobiological perspective, lymph nodes are not only critical sites for antigen presentation and immune priming, but also essential niches for the maintenance, expansion, and differentiation of stem-like T cells into effector cytotoxic T cells. In contrast, peripheral immune organs such as the spleen have limited capacity for “re-education” and clonal expansion of these T-cell subsets (42). Therefore, lymph nodes function not only as antigen convergence centers but also as key sites for T-cell lineage programming and sustained anti-tumor immune output, and are considered both a “training ground” and an “effector source” of systemic anti-tumor immunity (43). Accordingly, the structural integrity and immunological status of lymph nodes may directly influence the magnitude and durability of immunotherapy responses.

Against this background, the traditional oncological surgical concept of “extended dissection for metastasis control” is being re-examined in the era of immunotherapy. Importantly, the potential immunological implications of lymphadenectomy should be interpreted according to lymph node status. Metastatic lymph nodes, characterized by tumor cell infiltration and potential disruption of lymphoid architecture, remain critical targets for surgical removal to achieve oncological control. Although systematic removal of regional lymph nodes during radical surgery helps eliminate potential metastatic deposits, it may also simultaneously remove non-metastatic lymphoid tissues with potentially preserved immune functions. Therefore, the theoretical concern regarding the immunological impact of lymphadenectomy primarily applies to non-metastatic lymph nodes with preserved immune structures and potential functions in antigen presentation and T-cell priming. However, current preoperative imaging modalities, including computed tomography and endoscopic ultrasonography, cannot reliably distinguish metastatic from non-metastatic lymph nodes, particularly after neoadjuvant immunotherapy, when treatment-related fibrosis, inflammation, and immune-cell infiltration may mimic residual nodal disease. This diagnostic limitation represents a major barrier to implementing response-guided or immune-guided lymphadenectomy strategies.

The ATTRACTION-5 trial provides an opportunity to reconsider the interaction between surgery and perioperative immunotherapy. This study evaluated adjuvant immunotherapy after curative surgery but failed to demonstrate a survival benefit. Notably, the enrolled population was predominantly from Japan (48%), Korea (42%), and China (~10%), where standardized D2 or D2+ lymphadenectomy is routinely performed, with the average number of retrieved lymph nodes often exceeding 40. However, the absence of a survival benefit cannot be attributed to the extent of lymphadenectomy alone. The efficacy of immunotherapy is influenced by multiple treatment-related and biological factors, and the specific contribution of lymphadenectomy. These observations raise the hypothesis that the extent of lymph node dissection may potentially influence immune-related processes; however, this concept remains theoretical and lacks direct clinical validation in gastric cancer. Therefore, the unfavorable outcomes observed in perioperative immunotherapy trials, including KEYNOTE-585 and ATTRACTION-5, should not be interpreted as evidence against standard lymphadenectomy. Instead, they likely reflect the complex interplay of multiple factors, including inadequate biomarker-based patient selection, differences in treatment sequencing, variability in postoperative immunotherapy exposure, surgical stress-induced immune alterations, and heterogeneity in adjuvant treatment strategies. In LAGC, neoadjuvant therapy has become a standard treatment strategy, and the addition of immunotherapy has significantly improved pathological response rates and R0 resection rates. However, optimization of surgical approaches and lymph node dissection strategies has lagged behind. Current surgical planning is mainly based on pre-treatment clinical staging. In the context of significant downstaging induced by neoadjuvant immunotherapy, even achieving clinical complete response (cCR) or pathological complete response (pCR), it remains unclear whether standard D2 dissection based on initial staging is still necessary, and whether surgical extent should be adapted according to treatment response. High-quality evidence addressing these issues is still lacking.

A more challenging question is whether a “watch-and-wait” strategy, similar to that used in rectal cancer, could be applied to selected patients achieving cCR. This remains exploratory. A 2023 study from the University of California, San Francisco published in *Cell* further emphasized the critical role of lymph nodes in immunotherapy. In a head and neck squamous cell carcinoma model, the study showed that persistent regional lymph nodes serve as a key source for expansion and maintenance of anti-tumor T cells. The efficacy of immunotherapy depends, at least in part, on continuous activation and output of T cells within lymph nodes, rather than solely on immune cells within the primary tumor microenvironment (45). This finding suggests that systematic lymph node removal may inadvertently disrupt the immune amplification loop.

Reconsidering radical gastrectomy from this perspective, standard D2 lymphadenectomy, while ensuring oncological radicality, may also substantially disrupt the structural integrity of the gastric lymphatic immune microenvironment. Importantly, pivotal trials such as KEYNOTE-585 (11) and ATTRACTION-5 (29) did not standardize or stratify surgical procedures and lymph node dissection extent in detail, making it difficult to isolate the impact of surgical variability on immunotherapy efficacy and introducing potential confounding factors.

Therefore, in the era of immunotherapy, whether standardized D2 lymphadenectomy remains the optimal surgical strategy for LAGC requires re-evaluation. On one hand, D2 dissection remains fundamental for achieving R0 resection and accurate staging. On the other hand, excessive removal of non-metastatic lymph nodes may increase surgical morbidity and potentially compromise the long-term translational benefit of immunotherapy and postoperative quality of life.

Overall, immunotherapy combined with chemotherapy has significantly improved tumor regression and resection rates in LAGC, but its survival benefit has not been consistently translated. Optimization of perioperative therapy may depend not only on drug regimens but also on the interaction between surgical strategies and the immune ecosystem. Therefore, high-quality prospective studies are urgently needed to systematically evaluate the relationship between lymph node dissection extent, surgical approaches, and immunotherapy efficacy, and to determine whether an “immune-friendly” surgical strategy can be defined while maintaining oncological radicality.

Key scientific questions include how to accurately distinguish metastatic from functionally normal lymph nodes preoperatively, whether selective lymph node preservation based on immune status can be established, and whether preservation of partial lymph node function could enhance postoperative immunotherapy efficacy and improve long-term survival. Addressing these questions may fundamentally reshape the surgical paradigm for LAGC in the era of immunotherapy. The interactions among neoadjuvant immunotherapy, the dual roles of regional lymph nodes, and the potential oncological and immunological effects of lymphadenectomy are illustrated in **Figure 1**.

**Figure 1:**
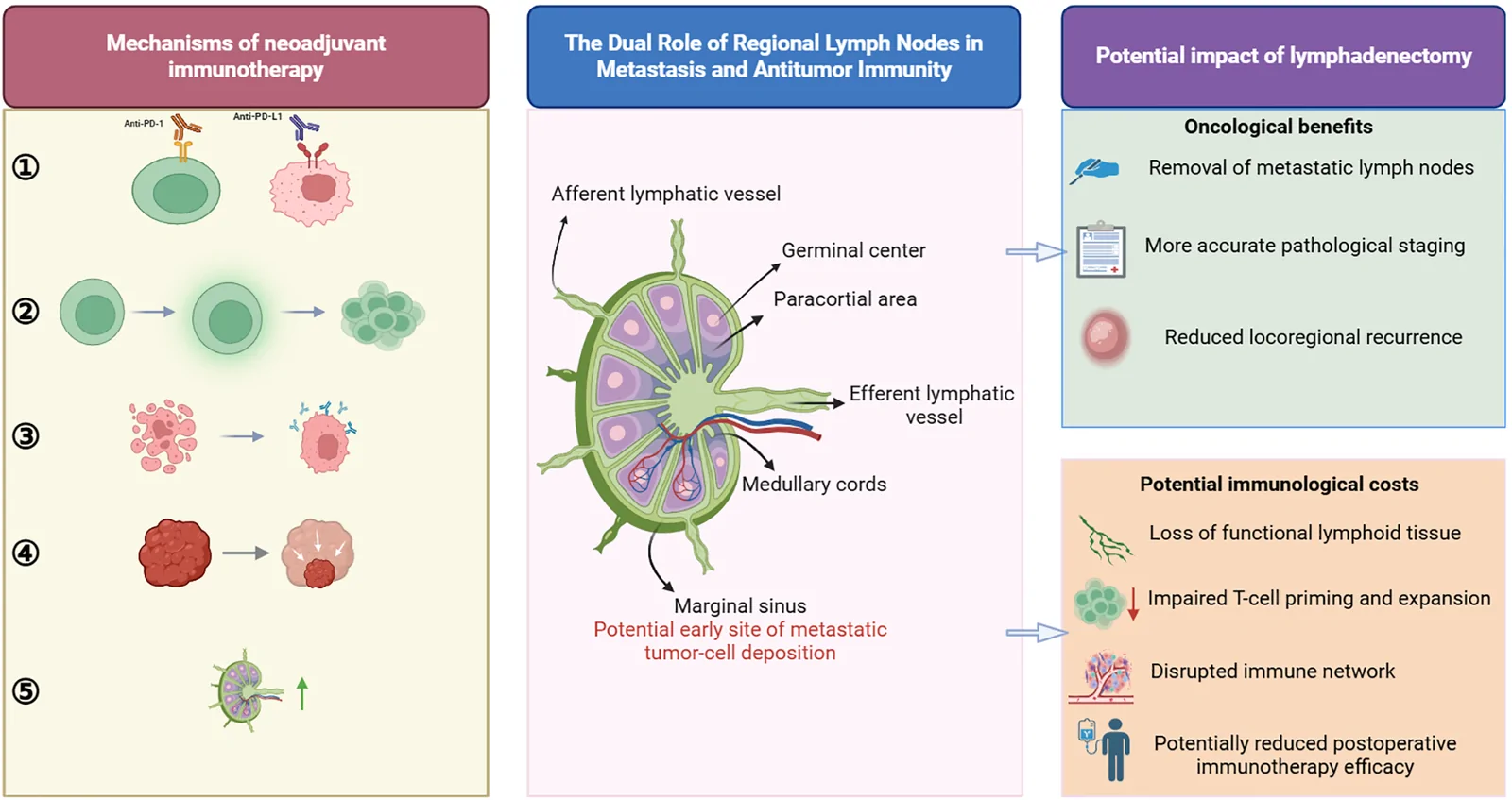
Interactions among neoadjuvant immunotherapy, regional lymph nodes, and lymphadenectomy in locally advanced gastric cancer. (The left panel sequentially illustrates five steps: ① PD-1/PD-L1 blockade, ② T-cell activation and clonal expansion, ③ immune-mediated tumor-cell killing and antigen release, ④ tumor regression, and ⑤ activation of regional antitumor immunity).

## 5 Neoadjuvant immunotherapy and surgical strategy

Neoadjuvant immunotherapy combined with chemotherapy has become an important direction in the perioperative management of locally advanced gastric cancer (LAGC). Its primary goals are to achieve tumor downstaging, increase pathological response rates, improve the likelihood of R0 resection, and ultimately enhance long-term survival. The decision of whether and when to proceed with surgery after perioperative immunotherapy depends on tumor response, the patient’s overall condition, and the expected survival benefit.

Based on treatment response, patients can be classified into radiological partial response (rPR), radiological complete response (rCR), clinical complete response (cCR), and pathological complete response (pCR). Among these, pCR is considered the most favorable endpoint; however, it can only be confirmed postoperatively, and accurate preoperative identification remains limited. Therefore, for most patients, radical surgery remains essential for pathological confirmation and durable tumor control.

Current evidence indicates that patients achieving rPR benefit significantly from surgical resection in terms of overall survival (OS) and event-free survival (EFS). The NEOSUMMIT-01 study further demonstrated that treatment-sensitive patients derive greater EFS benefit after radical surgery, supporting an active surgical approach in this population (46). For patients achieving rCR, the necessity of surgery remains controversial. Because rCR does not necessarily correspond to pCR, residual tumor cells may still be present despite radiological disappearance. Therefore, for patients with acceptable surgical risk, radical resection is still considered the most reliable strategy.

In recent years, with the substantial improvement in pathological response rates achieved by immunotherapy, whether patients with cCR can be managed using a “watch-and-wait” (W&W) strategy, similar to rectal cancer, has become an area of active investigation. However, there is currently no unified definition of cCR in gastric cancer. It generally requires radiological complete response, normalization of tumor markers, absence of distant metastasis, and sustained disease stability over time. For selected patients achieving cCR with high surgical risk, a W&W strategy may be theoretically feasible. However, existing evidence is limited to case reports and small cohort studies, and its safety and long-term efficacy remain unproven in prospective trials (47, 48).

In particular, for mismatch repair-deficient (dMMR) or microsatellite instability-high (MSI-H) gastric cancer, perioperative immunotherapy can achieve pCR rates of 60%–80%. In patients who achieve both cCR and circulating tumor DNA (ctDNA) negativity, non-operative management under strict surveillance may be worthy of further investigation. However, for patients who do not achieve cCR or who remain ctDNA-positive, radical surgery is still recommended (27, 49).

Molecular subtype represents an important determinant of response to neoadjuvant immunotherapy and may provide a foundation for future risk-adapted surgical strategies. MSI-H/dMMR gastric cancers demonstrate substantially higher sensitivity to immune checkpoint blockade compared with MSS/pMMR tumors, with markedly increased pathological response rates reported in clinical studies. Conversely, MSS/pMMR tumors generally exhibit lower sensitivity to immunotherapy and remain associated with a higher likelihood of residual disease after systemic treatment. HER2-positive gastric cancer represents another biologically distinct subgroup, in which immunotherapy combined with HER2-targeted therapy has shown promising activity. However, despite these differences in treatment sensitivity, current evidence remains insufficient to support molecular subtype-based modification of gastrectomy extent or D2 lymphadenectomy. At present, molecular classification should primarily serve as a tool for risk stratification, treatment selection, and prospective clinical trial design rather than routine alteration of surgical practice. Molecular subtype is an important determinant of response to neoadjuvant immunotherapy and may provide a basis for future risk-adapted surgical strategies. MSI-H/dMMR tumors generally show higher sensitivity to immune checkpoint inhibitors, whereas MSS/pMMR tumors have limited response and a higher risk of residual disease. HER2-positive tumors may benefit from combined immunotherapy and HER2-targeted therapy. However, current evidence does not support routine modification of gastrectomy extent or D2 lymphadenectomy based on molecular subtype. The potential implications of different molecular subtypes for current management and future research are summarized in **Table 3**.

**Table 3:** Molecular subtype-based stratification and future perspectives.

Molecular subtype	Treatment response characteristics	Current standard of care	Future research priorities
MSI-H/dMMR	High sensitivity to immune checkpoint inhibitors; higher pCR/cCR rates	Standard gastrectomy with D2 lymphadenectomy remains recommended	Explore response-guided strategies in prospective trials
MSS/pMMR	Limited immunotherapy sensitivity; higher risk of residual disease	Standard gastrectomy with D2 lymphadenectomy according to clinical stage standard	Identify predictive biomarkers and optimize combination therapies
HER2-positive	Potential benefit from immunotherapy plus HER2-targeted therapy; heterogeneous response	Surgical management based on oncological principles and multidisciplinary evaluation	Develop individualized perioperative strategies integrating HER2 and immune biomarkers

Beyond surgical indications, timing of surgery is another key issue in perioperative management. Although no direct evidence defines the optimal timing after immunotherapy, current studies and clinical practice generally suggest that surgery performed 4–6 weeks after the last treatment can balance tumor control and perioperative safety (50). For patients receiving anti-angiogenic agents, a drug-free interval of 2–4 weeks is usually recommended to reduce intraoperative bleeding and anastomotic complications. In addition, recovery from immune-related adverse events (irAEs) must be carefully assessed preoperatively to ensure surgical safety (6, 23).

Notably, multiple studies suggest that most immunotherapy-sensitive patients achieve significant tumor regression after 2–4 treatment cycles, and extending neoadjuvant therapy beyond this period does not provide additional survival benefit. Conversely, prolonged treatment may increase toxicity and delay surgery. Therefore, once surgical eligibility is established, unnecessary treatment prolongation should be avoided, and timely radical resection should be performed after comprehensive evaluation (10, 12, 51).

In the postoperative setting, the primary goal of adjuvant therapy is to eliminate micrometastatic disease, reduce recurrence risk, and prolong survival. Given the persistently high recurrence rate in LAGC, the concept of “perioperative full-course immunotherapy” has gained increasing attention. This strategy aims to maintain continuous antitumor immune pressure through both neoadjuvant and adjuvant phases. The rationale is that microscopic residual disease may persist after surgery, and surgical stress can induce transient immunosuppression; therefore, continued immunotherapy may help sustain and reinforce pre-established antitumor immune responses (52, 53).

In current clinical practice, adjuvant treatment strategies are increasingly individualized. For patients achieving pCR, with early-stage disease and without high-risk pathological features, shorter or de-escalated adjuvant therapy may be considered. In contrast, for patients without pCR, or those with lymph node metastasis, vascular invasion, or other high-risk features, continuation or intensification of the original regimen is generally recommended. In addition, circulating tumor DNA (ctDNA), as a key biomarker of minimal residual disease (MRD), is becoming an important tool for guiding adjuvant therapy decisions. Persistent postoperative ctDNA positivity indicates a higher risk of recurrence and may identify patients who benefit more from intensified adjuvant treatment (54).

Perioperative immunotherapy also requires careful management of immune-related adverse events (irAEs). irAEs may involve the skin, endocrine system, liver, lungs, and gastrointestinal tract, with an incidence of approximately 10%–30%, and grade 3–4 events accounting for about 5%–10%. Most mild to moderate irAEs can be managed with close monitoring while continuing treatment, whereas severe irAEs require interruption of immunotherapy and administration of corticosteroids. In the perioperative setting, special attention is needed to differentiate irAEs from postoperative complications to avoid delayed or inappropriate management. Overall, a multidisciplinary team approach is essential to ensure the safe implementation of perioperative immunotherapy.

## 6 Discussion

The introduction of immune checkpoint inhibitors into the perioperative treatment of locally advanced gastric cancer has substantially improved pathological response and tumor downstaging. Several recent trials have reported higher rates of pathological complete response and major pathological response with immunotherapy-based regimens than with chemotherapy alone. However, improvements in pathological response have not consistently translated into long-term survival benefits. Previous evidence suggests that more extensive lymphadenectomy may increase postoperative morbidity and perioperative mortality, while chemotherapy may also exert paradoxical biological effects that potentially promote tumor relapse and metastatic progression (55, 56). These findings indicate that pathological regression alone may be insufficient to define the optimal surgical strategy and that the interaction among treatment response, residual nodal disease, surgical radicality, and postoperative therapy requires further investigation.

One of the major challenges after neoadjuvant immunotherapy is the accurate assessment of residual disease. Radiological regression of the primary tumor does not necessarily indicate complete pathological response, and the response of regional lymph nodes may differ from that of the primary lesion. Residual tumor cells may remain within lymph nodes despite substantial regression of the gastric lesion, whereas inflammatory enlargement, fibrosis, necrosis, or immune-cell infiltration may mimic persistent nodal metastasis. Consequently, current imaging modalities cannot reliably distinguish residual metastatic nodes from treatment-related immune or inflammatory changes. This uncertainty limits the use of radiological response alone to guide modification of lymphadenectomy.

Regional lymph nodes also have dual oncological and immunological roles. From an oncological perspective, they are common sites of early metastatic spread, and adequate lymphadenectomy contributes to regional disease control, pathological staging, and postoperative risk stratification. From an immunological perspective, regional lymph nodes are important sites for antigen presentation, T-cell priming, clonal expansion, and maintenance of antitumor immune responses. Their removal may therefore theoretically alter regional immune networks and influence subsequent responses to immunotherapy. Nevertheless, current evidence supporting this hypothesis is mainly translational and indirect. No prospective clinical study in gastric cancer has demonstrated that preservation of regional lymph nodes improves immunotherapy efficacy or long-term survival.

Accordingly, standard D2 lymphadenectomy should remain the recommended surgical approach for resectable locally advanced gastric cancer after neoadjuvant immunotherapy. Treatment response, radiological downstaging, or apparent clinical complete response should not currently be used as independent reasons to reduce the standard D2 field. At the same time, more extensive lymphadenectomy beyond standard D2 boundaries should not be performed routinely without clear anatomical or oncological indications. The current clinical priority is therefore not simple de-escalation, but risk-adapted surgical optimization that preserves oncological adequacy while avoiding unnecessary extended dissection.

## 7 Future directions

With the development of precision oncology and multidisciplinary decision-making, future optimization of lymphadenectomy after neoadjuvant immunotherapy for locally advanced gastric cancer should focus on several key areas.

First, risk-adapted and response-informed optimization of lymphadenectomy should be further investigated. At present, the extent of lymph node dissection is still determined mainly by the type of gastrectomy, tumor location, and pretreatment clinical stage. In the future, multidimensional information—including tumor location, pretreatment nodal distribution, radiological response, pathological regression patterns of both the primary tumor and lymph nodes, molecular subtype, and dynamic changes in circulating tumor DNA—may improve the assessment of residual disease and recurrence risk. Artificial intelligence-assisted analysis integrating contrast-enhanced CT, endoscopic ultrasonography, PET/CT, and radiomics may also help distinguish residual metastatic lymph nodes from treatment-related inflammatory or immune-reactive changes. However, these approaches cannot yet accurately define the anatomical extent of lymphadenectomy and should not replace standard D2 lymphadenectomy.

Second, an appropriate balance should be sought between oncological clearance and preservation of regional lymph node-associated immune function. Regional lymph nodes are not only important sites of gastric cancer metastasis but also essential sites for antigen presentation, T-cell priming, clonal expansion, and maintenance of antitumor immunity. Removal of non-metastatic but immunologically functional lymphoid tissue may theoretically alter regional immune networks and subsequent responses to immunotherapy. Nevertheless, this hypothesis has not yet been validated in prospective clinical studies of gastric cancer. Therefore, an immune-friendly approach to lymphadenectomy should not currently be interpreted as routine reduction of the standard D2 field, but rather as avoidance of unnecessary extended lymphadenectomy in the absence of clear oncological indications.

Third, multidisciplinary team-based surgical decision-making should be further strengthened. The timing of surgery, extent of gastric resection, scope of lymphadenectomy, and postoperative treatment strategy after neoadjuvant immunotherapy should be jointly evaluated by surgeons, medical oncologists, radiologists, pathologists, endoscopists, nutrition specialists, anesthesiologists, and other relevant specialists. Decision-making should comprehensively consider pretreatment stage, tumor location, treatment response, immune-related adverse events, the patient’s general condition, operative risk, and the feasibility of achieving R0 resection. Such an approach may help reduce both inadequate nodal clearance and unnecessary extension of lymphadenectomy.

Fourth, high-quality prospective and translational studies are required. Future studies should stratify patients according to pretreatment nodal stage, tumor location, molecular subtype, concordance between primary-tumor and nodal responses, pathological response, and residual nodal burden. In addition to conventional outcomes such as perioperative morbidity, recurrence, disease-free survival, and overall survival, these studies should evaluate nodal pathological regression, the distribution of positive lymph nodes, immune-cell composition, ctDNA dynamics, recurrence patterns, and quality of life. At present, priority should be given to prospective cohort studies and mechanistic investigations. Only after reliable patient-selection criteria and oncological safety have been established should response-guided reduction or modification of lymphadenectomy be further evaluated in clinical trials.

Fifth, Establishing a prospective validation framework for individualized lymphadenectomy. To translate the concept of response-guided or immune-informed lymphadenectomy into clinical practice, prospective validation studies are required before any modification of standard D2 lymphadenectomy can be considered. A potential research framework would include a prospective observational cohort study enrolling patients with resectable locally advanced gastric cancer treated with neoadjuvant immunotherapy followed by curative gastrectomy. Serial ctDNA assessment before, during, and after systemic treatment could be integrated with radiological response evaluation, molecular subtype classification, and detailed station-specific pathological assessment of lymph nodes. Furthermore, characterization of the tumor immune microenvironment, including immune-cell composition and spatial immune profiling, may provide additional biological insights into treatment response and lymph node-associated immune function. The primary endpoint of such studies could be 3-year disease-free survival, while secondary endpoints may include overall survival, recurrence patterns, pathological nodal regression, surgical morbidity, quality of life, and biomarker-response concordance. Such prospective investigations may help identify highly selected patients, such as those with MSI-H/dMMR tumors, sustained ctDNA negativity, or confirmed clinical complete response, who may be candidates for future trials evaluating individualized lymphadenectomy strategies.

## 8 Conclusion

Neoadjuvant immunotherapy has reshaped the perioperative management of locally advanced gastric cancer by improving pathological response and tumor downstaging. However, current evidence does not support routine reduction of gastric resection or standard D2 lymphadenectomy based solely on treatment response. Regional lymph nodes have both metastatic and immunological functions, but the clinical value of preserving immunologically functional nodes remains uncertain. Until reliable methods can distinguish residual metastatic nodes from treatment-related immune changes, standard D2 lymphadenectomy based on pretreatment stage, tumor location, and surgical anatomy should remain the preferred approach.

Future studies should investigate whether response-informed or immune-guided lymphadenectomy strategies can be safely implemented in highly selected populations. Biomarker-stratified prospective trials incorporating molecular characteristics such as MSI-H/dMMR status, dynamic biomarkers such as ctDNA, and standardized definitions of clinical complete response are required before lymphadenectomy de-escalation can be considered. At present, reduction of lymph node dissection should remain investigational and should only be evaluated within prospective clinical trials.
